# Evaluating the Predictive Accuracy of Socioeconomic Metrics on Heart Failure Risk and Outcomes in Maryland

**DOI:** 10.7759/cureus.69474

**Published:** 2024-09-15

**Authors:** Oluwasegun A Akinyemi, Mojisola E Fasokun, Oluranti Babalola, Oreoluwa Adubi, Oluwatayo J Awolumate, Nnaemeka Agunwa, Funmilola Belie, Seun A Ikugbayigbe, Kakra Hughes, Miriam Micheal

**Affiliations:** 1 Health Policy and Management, University of Maryland School of Public Health, College Park, USA; 2 Surgery, Howard University College of Medicine, Washington DC, USA; 3 Epidemiology and Public Health, University of Alabama at Birmingham, Birmingham, USA; 4 Social Work, University of South Carolina, Columbia, USA; 5 Internal Medicine, Ross University School of Medicine, Miramar, USA; 6 Internal Medicine, Howard University College of Medicine, Washington DC, USA; 7 Public Health, Western Illinois University, Macomb, USA; 8 Public Health, Southern Connecticut State University, New Haven, USA; 9 Biological Sciences, Eastern Illinois University, Charleston, USA; 10 Internal Medicine, University of Maryland School of Medicine, Baltimore, USA

**Keywords:** atrial fibrillation, cardiac arrest, distressed communities index, heart failure, mortality, neighborhood poverty, predictive modelling, prolonged hospital stay, social determinants of health

## Abstract

Introduction

Annually, a significant number of Americans are hospitalized due to heart failure (HF), marking it as an important contributor to morbidity and mortality. It also poses a substantial financial burden and leads to considerable losses in productivity. Socioeconomic disparities may intensify the risk of hospital admissions following HF and worsen patient outcomes.

Objective

This study investigates the predictive accuracy of different socioeconomic metrics on the risk and outcomes of HF in Maryland.

Methodology

To evaluate the predictive accuracy of various socioeconomic metrics on the risk of HF, we utilized data from the Maryland State Inpatient Database. Our retrospective analysis covered hospital admissions for HF from 2016 to 2020, correlating these with poverty indicators derived from U.S. Census data at the zip code level with socioeconomic metrics like race/ethnicity, insurance, household median income, and neighborhood distress (Distressed Communities Index (DCI)). Multivariate logistic regression models adjusted for confounders and isolated the impact of socioeconomic factors.

Result

During the study period, a total of 389,220 cases of HF were reported in the Maryland State Inpatient Database (SID). The majority of these patients were White individuals (56.8%) and female (51.1%), with a median age of 73 years (interquartile range (IQR) 62-82 years). The in-hospital mortality rate was 5.1%, while rates of atrial fibrillation, cardiac arrest, and prolonged hospital stay were 34.4%, 0.3%, and 48.4%, respectively. The studied socioeconomic metrics showed varying predictive power for the risk of HF-related admissions and selected outcomes, with the highest predictive accuracy for neighborhood distress on the risk of HF (AUC = 0.53, 95% CI 0.530-0.532), atrial fibrillation (AUC = 0.479, 95% CI 0.477-0.480), cardiac arrest (AUC = 0.511, 95% CI 0.498-0.525), prolonged hospital stays (AUC = 0.531, 95% CI 0.530-0.532), and mortality (AUC = 0.499, 95% CI 0.496-0.502).

Conclusions

The Distressed Communities Index demonstrates significant predictive power for assessing the risk of hospital admissions following HF and outcomes among individuals with HF, exceeding factors like insurance, race/ethnicity, and household median income.

## Introduction

Heart failure (HF) remains a critical public health challenge in the United States, contributing significantly to morbidity and mortality [1]. Annually, over 500,000 Americans are hospitalized due to heart failure, underscoring its role as a major contributor to healthcare utilization [2]. The age-adjusted rate of heart disease deaths has increased each year since 2020 [3,4]. Financially, HF imposes a substantial burden, costing U.S. taxpayers more than $30 billion annually and resulting in considerable productivity losses [5,6]. This condition not only affects the health of individuals but also places a significant strain on the healthcare system and the economy.

Socioeconomic factors play a crucial role in the prevalence and outcomes of heart failure. Metrics such as race/ethnicity, insurance type, and income levels provide insight into the disparities that exist in healthcare access and outcomes [7-9]. The median household income of a patient's zip code, state quartile income levels, and the Distressed Communities Index (DCI) are commonly used to measure socioeconomic status and explore its impact on health disparities [10-12]. These metrics offer valuable perspectives on how economic and social conditions influence health outcomes across different communities.

Studies have shown that socioeconomic factors can significantly influence the incidence and prognosis of heart failure [13,14]. For instance, lower socioeconomic status has been linked to higher rates of hospitalization and worse health outcomes, including higher mortality rates among heart failure patients [13-15]. The disparities highlighted by these socioeconomic measures suggest that certain populations are at higher risk and may not receive the necessary care to effectively manage their conditions.

Given the profound impact of socioeconomic factors on heart failure, it is essential to identify which metrics are the most predictive of heart failure prevalence and outcomes. This study aims to evaluate the predictive accuracy of various socioeconomic indicators, such as insurance status, income level, and community distress, on the prevalence and short-term outcomes of heart failure in Maryland. Understanding which socioeconomic metrics are most impactful could guide targeted interventions and policies to reduce the burden of heart failure across diverse populations.

## Materials and methods

Data source and study population

The study was conducted at the Clive O Callender Outcomes Research Center of the Howard University College of Medicine, Washington DC, USA. Our study utilized the Maryland State Inpatient Database (SID) for the years 2016 to 2020. This comprehensive dataset encompasses all hospital admissions in the state, providing detailed information on the demographic, clinical, and socioeconomic characteristics of patients. The study population included individuals aged 18 and older with a diagnosis of heart failure, as well as a cohort without this diagnosis, to compare selected outcomes between the two groups. Heart failure diagnoses were identified using the International Classification of Diseases (ICD-10) diagnosis codes. 

Outcome measures

The outcomes measured in this study included the prevalence of heart failure during admission, in-hospital mortality, prolonged hospital stays (defined as stays longer than the median stay duration for the cohort), rates of atrial fibrillation, and cardiac arrest during the hospitalization period.

Independent variables and covariates

The primary independent variables in this study were socioeconomically focused and included race/ethnicity, insurance type, median household income, and the Distressed Communities Index (DCI). The data in the Healthcare Cost and Utilization Project (HCUP) defined the race as White, Black, Hispanic, Asian/Pacific Islander, Native American, and Other populations. Race/ethnicity was categorized into White, Black, Hispanic, Non-Hispanic Asian American/Pacific Islander, Native American, and Other populations. Insurance types were categorized as Medicare, Medicaid, Private, Self-Pay, and Other. Median household income was analyzed at both the state and national quartiles for each patient's zip code. The DCI, which measures economic distress at the zip code level, was also included as a key socioeconomic metric.

Covariates

In addition to the primary independent variables, several covariates were considered to adjust for potential confounders. These included age, stratified into groups (<45 years, 45-64 years, and >65 years), gender, and various health conditions such as hypertension, diabetes, alcohol use, smoking, depression, bipolar disorder, and obesity. The inclusion of weekend admission status provided additional adjustment for potential variations in staffing and hospital operational factors during these times.

Statistical analysis

To assess the effects of socioeconomic indicators on heart failure outcomes, we conducted multivariable logistic regression analyses, adjusting for demographic and health-related covariates.

The predictiveness of each socioeconomic metric was assessed through the development of margin plots. These plots visualized the adjusted probabilities of each outcome across different levels of the socioeconomic indicators, providing a clear and interpretive graphical representation of the data.

Model performance evaluation

The discriminative ability of each logistic regression model was quantified by calculating the area under the receiver operating characteristic (ROC) curve (AUC). This measure is essential in evaluating the model's effectiveness in distinguishing between patients with and without the outcomes of interest at various decision thresholds.

By integrating a comprehensive analysis of socioeconomic metrics with clinical outcomes in heart failure, this methodology aims to provide insights that could potentially guide future healthcare policies and interventions aimed at mitigating the impact of socioeconomic disparities on heart failure outcomes in Maryland.

## Results

Association of baseline characteristics with heart failure-related admissions

In Table 1, the individual's baseline characteristics, such as age, sex, and race/ethnicity, significantly influenced the likelihood of heart failure-related admissions. Patients aged over 65 accounted for 271,633 (69.8%) of heart failure admissions, compared to 781,748 (31.3%) in the non-heart failure group. Females comprised 198,842 (51.1%) of heart failure cases compared to 1,441,102 (57.8%) in those without heart failure, indicating a lower incidence of admissions among females (p < 0.01). Black patients made up 146,626 (37.9%) of heart failure admissions versus 802,980 (32.5%) in the non-heart failure cohort, while Hispanic and Asian/Pacific Islander patients had a lower representation among heart failure admissions. 

**Table 1:** Baseline characteristics of individuals with heart failure (Maryland State Inpatient Database 2016-2020) Chi-square (χ²) tests were used to calculate the p-values for categorical variables. Test statistics are provided in the corresponding column. A p-value < 0.01 indicates statistical significance. HF: heart failure

Variables	Total Population (N=2,884,598)	Heart Failure (N=389,220)	Non-HF Admissions (N=2,495,378)	Statistics	P-value
Age (Years)				χ2 =2.7e+05	<0.01
18-44yrs	1,093,791 (37.9%)	15,262 (3.9%)	1,078,529 (43.2%)		
45-65yrs	737,426 (25.6%)	102,325 (26.3%)	635,101 (25.5%)		
>65yrs	1,053,381 (36.5%)	271,633 (69.8%)	781,748 (31.3%)		
Female	1,639,944 (56.9%)	198,842 (51.1%)	1,441,102 (57.8%)	χ2 =6.1e+03	<0.01
Race/Ethnicity				χ2 =2.3e+04	<0.01
White	1,551,208 (54.3%)	219,657 (56.8%)	1,331,551 (53.9%)		
Black	949,606 (33.3%)	146,626 (37.9%)	802,980 (32.5%)		
Hispanics	191,712 (6.7%)	8,182 (2.1%)	183,530 (7.4%)		
Asians/Pacific Islander	84,733 (3.0%)	5,801 (1.5%)	78,932 (3.2%)		
Native Americans	6,451 (0.2%)	718 (0.2%)	5,733 (0.2%)		
Others	72,538 (2.5%)	5,583 (1.4%)	66,955 (2.7%)		
Household median income				χ2 =6.5e+03	<0.01
Quartile I	354,213 (12.4%)	58,829 (15.3%)	295,384 (12.0%)		
Quartile II	359,318 (12.6%)	56,118 (14.5%)	303,200 (12.3%)		
Quartile III	885,300 (31.0%)	119,086 (30.8%)	766,214 (31.0%)		
Quartile IV	1,260,175 (44.1%)	152,285 (39.4%)	1,107,890 (44.8%)		
Distressed Communities Index				χ2 =2.7e+03	<0.01
Prosperous	385,977 (25.4%)	48,483 (22.6%)	337,494 (25.9%)		
Comfortable	393,167 (25.9%)	54,391 (25.3%)	338,776 (26.0%)		
Mid-tier	343,772 (22.7%)	46,875 (21.8%)	296,897 (22.8%)		
At-risk	174,274 (11.5%)	27,746 (12.9%)	146,528 (11.3%)		
Distressed	220,148 (14.5%)	37,181 (17.3%)	182,967 (14.1%)		
Insurance types				χ2 =2.3e+05	<0.01
Medicare	1,165,035 (40.4%)	294,013 (75.6%)	871,022 (34.9%)		
Medicaid	706,268 (24.5%)	40,851 (10.5%)	665,417 (26.7%)		
Private	881,817 (30.6%)	44,738 (11.5%)	837,079 (33.6%)		
Self-pay	41,586 (1.4%)	2,554 (0.7%)	39,032 (1.6%)		
No charge	7,147 (0.3%)	139 (0.04%)	7,008 (0.3%)		
Other	81,764 (2.8%)	6,811 (1.8%)	74,953 (3.0%)		
Lifestyle behaviours					
Depression	407,152 (14.1%)	62,480 (16.1%)	344,672 (13.8%)	χ2 =1.4e+03	<0.01
Alcohol abuse	114,300 (4.0%)	10,450 (2.7%)	103,850 (4.2%)	χ2 =1.9e+03	<0.01
Drug abuse	365 (0.01%)	<10 (0.00%)	357 (0.01%)	χ2 =39.94	<0.01
Smoking	402,206 (13.9%)	49,930 (12.8%)	352,276 (14.1%)	χ2 =466.18	<0.01
Comorbidities					
Hypertension	929,786 (32.3%)	53,421 (13.7%)	876,365 (35.1%)	χ2 =7.1e+04	<0.01
Diabetes	487,404 (16.9%)	146,692 (37.7%)	340,712 (13.7%)	χ2 =1.4e+05	<0.01
Bipolar disorder	116,758 (4.1%)	9,729 (2.5%)	107,029 (4.3%)	χ2 =2.8e+03	<0.01
Anxiety disorder	404,730 (14.0%)	57,962 (14.9%)	346,768 (13.9%)	χ2 =276.59	<0.01
Personality disorder	16,922 (0.6%)	607 (0.2%)	16,315 (0.7%)	χ2 =1.4e+03	<0.01
Dementia	204,820 (7.1%)	50,418 (13.0%)	154,402 (6.2%)	χ2 =2.3e+04	<0.01
Obesity	518,110 (18.0%)	110,192 (28.3%)	407,918 (16.4%)	χ2 =3.3e+04	<0.01

Socioeconomic factors and heart failure-related admissions

Socioeconomic factors, such as the DCI, insurance types, and household median income quintiles were significantly associated with heart failure-related admissions. Patients from distressed communities accounted for 37,181 (17.3%) of heart failure cases, compared to 182,967 (14.1%) of non-heart failure patients (p < 0.01). Medicare insured the majority of heart failure admissions, 294,013 (75.6%), followed by Medicaid, 40,851 (10.5%). Income also influenced admissions, with 58,829 (15.3%) heart failure patients in the lowest income quartile compared to 295,384 (12.0%) in the non-heart failure group. 

Association of comorbidities and lifestyle factors with heart failure-related admissions

Comorbidities and lifestyle factors also showed significant associations with heart failure-related admissions. Diabetes was prevalent in 146,692 (37.7%) of heart failure patients, compared to 340,712 (13.7%) without heart failure (p < 0.01), indicating a strong link between diabetes and heart failure risk. Similarly, obesity was observed in 110,192 (28.3%) of heart failure admissions versus 407,918 (16.4%) of non-heart failure cases, suggesting its contribution to heart failure risk (p < 0.01). Depression and dementia were also more common among heart failure patients, with 62,480 (16.1%) and 50,418 (13.0%) of heart failure patients having these comorbidities, respectively (p < 0.01). 

Table 2 compares the rates of selected complications, including atrial fibrillation, cardiac arrest, in-hospital mortality, and prolonged hospitalization, between patients admitted for heart failure and those admitted for non-heart failure conditions. 

**Table 2:** Association of comorbidities and lifestyle factors with heart failure-related admissions Chi-square (χ²) tests were used to calculate the p-values for categorical variables. Test statistics are provided in the corresponding column. A p-value < 0.01 indicates statistical significance.

Complications	Heart Failure (n=389,220)	No Heart Failure (n=2,495,378)	Statistics	p-value
Atrial fibrillation	133,698 (34.5%)	177,783 (7.1%)	χ2 =2.6e+05	< 0.01
Cardiac arrest	1,192 (0.3%)	1,522 (0.1%)	χ2 =2.2e+03	< 0.01
In-hospital mortality	19,790 (5.1%)	41,604 (1.7%)	χ2 =1.9e+04	< 0.01
Prolonged hospitalization	188,342 (48.4%)	683,925 (27.4%)	χ2 =7.0e+04	< 0.01

The analysis revealed that patients admitted for heart failure experienced significantly higher rates of complications compared to those admitted for non-heart failure conditions. Atrial fibrillation was prevalent in 133,698 (34.5%) heart failure patients, a stark contrast to the 177,783 (7.1%) observed in non-heart failure patients (p < 0.01), indicating a strong association between heart failure and arrhythmias. Cardiac arrest occurred in 1,192 (0.3%) of heart failure admissions compared to 1,522 (0.1%) in non-heart failure cases (p < 0.01), further underscoring the heightened cardiovascular risk. In-hospital mortality rates were notably higher among heart failure patients, at 19,790 (5.1%) versus 41,604 (1.7%) for those without heart failure (p < 0.01), highlighting the severe prognosis associated with heart failure. Additionally, prolonged hospitalization was more common in heart failure patients, with 188,342 (48.4%) experiencing extended stays compared to 683,925 (27.4%) non-heart failure admissions (p < 0.01). 

Table 3 presents the association of race/ethnicity, Distressed Communities Index, household median income, and insurance types with selected outcomes, including risk of heart failure-related admissions, cardiac arrest following heart failure admissions, in-hospital mortality, atrial fibrillation, and prolonged hospital stays after heart failure admissions.

**Table 3:** Association of the DCI, race/ethnicity, insurance types, and household median income quintiles with heart failure outcomes * indicates statistical significance. DCI: Distressed Communities Index

	Heart Failure	Atrial Fibrillation	Cardiac Arrest	Mortality	Prolonged Hospital Stay
	OR (95% CI)	OR (95% CI)	OR (95% CI)	OR (95% CI)	OR (95% CI)
Distressed Communities Index					
Prosperous	Reference	Reference	Reference	Reference	Reference
Comfortable	1.08 (1.06-1.09)*	0.99 (0.96-1.02)	1.02 (0.82-1.27)	1.09 (1.03-1.16)*	1.04 (1.01-1.07)*
Mid-tiered	1.08 (1.06-1.10)*	0.96 (0.93-0.99)*	0.85 (0.64-1.14)	1.17 (1.09-1.26)*	1.01 (0.98-1.05)
At-risk	1.21 (1.17-1.24)*	1.00 (0.96-1.05)	0.71 (0.48-1.05)	1.17 (1.06-1.30)*	1.08 (1.04-1.13)*
Distressed	1.12 (1.08-1.17)*	1.04 (0.97-1.11)	0.76 (0.46-1.25)	1.25 (1.09-1.44)*	1.15 (1.08-1.22)*
Race/Ethnicity					
White	Reference	Reference	Reference	Reference	Reference
Black	1.34 (1.32-1.35)*	0.63 (0.62-0.65)*	0.97 (0.82-1.15)	0.91 (0.87-0.95)	1.02 (1.00-1.04)*
Hispanic	0.68 (0.66-0.70)*	0.67 (0.63-0.72)*	1.04 (0.66-1.64)	0.99 (0.87-1.13)	1.02 (0.97-1.09)
Asian or Pacific Islander	0.82 (0.78-0.85)*	0.68 (0.63-0.73)*	2.10 (1.41-3.13)*	1.31 (1.16-1.49)*	1.05 (0.98-1.13)
Native American	1.08 (0.95-1.22)	0.67 (0.54-0.84)*	1.00 (empty)	0.63 (0.37-1.08)	1.12 (0.92-1.38)
Others	0.90 (0.85-0.94)*	0.77 (0.71-0.83)*	0.83 (0.44-1.55)	1.27 (1.11-1.46)*	1.09 (1.01-1.17)*
Insurance					
Self-pay	Reference	Reference	Reference	Reference	Reference
Medicare	1.55 (1.46-1.66)*	1.23 (1.08-1.41)*	0.53 (0.26-1.09)	0.89 (0.67-1.18)	1.30 (1.16-1.45)*
Medicaid	1.08 (1.01-1.15)*	1.00 (0.88-1.14)	0.80 (0.39-1.65)	0.98 (0.74-1.31)	1.38 (1.23-1.55)*
Private insurance	0.69 (0.65-0.74)*	1.06 (0.93-1.22)	1.11 (0.54-2.28)	1.07 (0.81-1.42)	1.20 (1.07-1.34)*
No charge	0.25 (0.19-0.31)*	0.87 (0.51-1.49)	-	1.40 (0.54-3.63)	1.92 (1.22-3.00)*
Others	0.91 (0.85-0.98)*	1.22 (1.05-1.42)*	0.46 (0.17-1.20)	3.36 (2.51-4.50)*	1.14 (1.00-1.30)*
Household median income					
Quartile I	Reference	Reference	Reference	Reference	Reference
Quartile II	0.90 (0.88-0.93)*	1.16 (1.10-1.22)*	0.97 (0.68-1.41)	0.94 (0.84-1.05)	0.99 (0.94-1.03)
Quartile III	0.87 (0.84-0.91)*	1.19 (1.12-1.26)*	0.65 (0.41-1.02)	1.07 (0.94-1.22)	1.00 (0.95-1.06)
Quartile IV	0.81 (0.78-0.84)*	1.20 (1.12-1.28)*	0.67 (0.41-1.09)	1.20 (1.04-1.38)*	0.95 (0.89-1.00)

Risk of heart failure

The DCI indicated a gradient of increasing odds for heart failure from more prosperous to more distressed zones. Specifically, individuals from "at-risk" areas had the highest odds (OR = 1.21, 95% CI 1.17-1.24) compared to the prosperous reference group, followed by "distressed" (OR = 1.12, 95% CI 1.08-1.17). 

In terms of race/ethnicity, compared to White patients, Black patients had 34% increased odds of heart failure-related admissions (OR = 1.34, 95% CI 1.32-1.35). Compared to the reference group, Hispanic patients (OR = 0.68, 95% CI 0.66-0.70) and Asian or Pacific Islander patients (OR = 0.82, 95% CI 0.78-0.85) experienced lower odds of heart failure. 

Insurance type also played a significant role, with Medicare patients showing 55% higher odds of heart failure compared to patients in the self-pay or uninsured category (OR = 1.55, 95% CI 1.46-1.66). Conversely, patients with private insurance had reduced odds (OR = 0.69, 95% CI 0.65-0.74). 

Socioeconomic status, as measured by the median household income quartiles, showed that individuals in higher income quartiles had progressively lower odds of heart failure compared to those in the lowest income quartile. Those in the highest income quartile experienced a 19% reduction in odds of heart failure compared to the reference group, which is individuals in the lowest income quartile (OR = 0.81, 95% CI 0.78-0.84). 

Mortality following heart failure

Compared to individuals from the wealthiest neighborhoods, residents of the distressed zones experienced significantly higher odds of mortality (OR = 1.25, 95% CI 1.09-1.44). Individuals who are residents of the comfortable, mid-tier, and at-risk areas also experienced higher odds of mortality compared to the reference group. Interestingly, Black patients experienced a marginal 9% lower odds of death compared to White patients with heart failure (OR = 0.91, 95% CI 0.87-0.95), while Asian/Pacific Islander individuals recorded 31% higher odds of mortality following heart failure compared to the reference group (OR = 1.31, 95% CI 1.16-1.49). There was no significant difference between White and Hispanic individuals mortality odds (OR = 0.99, 95% CI 0.87-1.13). 

There were no significant differences in mortality odds between the different insurance groups, and the reference was individuals with self-pay or uninsured status in this study. Interestingly, individuals in the highest income quartile experienced 20% higher odds of mortality compared to those in the lowest quartiles (OR = 1.20, 95% CI 1.04-1.38). 

Atrial fibrillation 

The multivariate analysis of factors associated with atrial fibrillation among patients admitted with heart failure showed that household median income and race were associated with the development of AF among individuals with heart failure. While residents of the distressed neighborhoods experienced a marginal 4% higher odds of AF compared to the reference group, this was not statistically significant (OR = 1.04, 95% CI 0.97-1.11). The DCI did not show a significant association with AF across different community distress levels, with only the 'mid-tiered' group experiencing a marginal 4% decreased odds compared to the reference (OR = 0.96, 95% CI 0.93-0.99). 

Compared to White patients, Black (OR = 0.63, 95% CI 0.62-0.65), Hispanic (OR = 0.67, 95% CI 0.63-0.72), Asian or Pacific Islander (OR = 0.68, 95% CI 0.63-0.73), and Native American (OR = 0.67, 95% CI 0.54-0.84) individuals all had significantly lower odds of AF. 

Regarding insurance type, Medicare recipients had 23% higher odds of AF compared to self-pay/uninsured (OR = 1.23, 95% CI 1.08-1.41), while no significant differences were found for other types. 

Household income quartiles showed higher odds of AF for higher income levels, with quartile IV exhibiting the highest odds (OR = 1.22, 95% CI 1.12-1.28). 

Cardiac arrest 

The logistic regression analysis for cardiac arrest among heart failure patients demonstrated marginal associations with the socioeconomic and demographic variables studied. While individual residents in the distressed communities have a 24% reduction in the odds of experiencing a cardiac arrest during a heart failure-related admission compared to their counterparts from the richest neighborhoods, this difference was not statistically significant. All DCI quintiles did not significantly predict cardiac arrest, with the odds ratios close to one across all categories, indicating minimal effect compared to the reference group. 

Race and insurance type showed limited influence. Asian or Pacific Islander individuals had 2.10 higher odds of cardiac arrest compared to White patients (OR = 2.10, 95% CI 1.41-3.13). Other racial groups and insurance types did not significantly predict the odds of cardiac arrest, with no clear patterns observed. 

The income quartile showed a non-significant trend towards lower odds of cardiac arrest in higher income quartiles compared to the lowest quartile. However, these results were not statistically robust. 

Prolonged hospital stays 

The multivariate analysis of factors associated with prolonged hospital stays among heart failure patients revealed that socioeconomic factors and race/ethnicity particularly significantly influenced hospital stay durations. Patients from distressed communities experienced 15% higher odds of a prolonged stay compared to those from prosperous zones (OR = 1.15, 95%CI 1.08-1.22). Also, individuals who reside in comfortable and at-risk areas have higher odds of experiencing prolonged hospital stays compared to the residents of prosperous neighborhoods. Similarly, Black patients experienced a marginal 2% higher odds of a prolonged hospital stay when compared to White patients (OR = 1.02, 95% CI 1.00-1.04). Individuals with self-pay or uninsured have the lowest odds of experiencing a prolonged hospital stay. At the same time, socioeconomic status, represented by different income quartiles, did not show a consistent impact on prolonged hospital stays, with no significant effects observed across higher income quartiles. 

Table 4 displays the relative predictive power of the DCI, race/ethnicity, insurance types, and household median income quintiles in predicting the risk of heart failure-related admissions and outcomes.

**Table 4:** Relative predictive power of DCI, race/ethnicity, insurance, and household median income on heart failure risk and outcomes *,p <0.05 DCI: Distressed Communities Index

	Heart Failure	Atrial Fibrillation	Cardiac Arrest	Mortality	Prolonged Hospital Stay
Variable	Area (95% CI)	Area (95% CI)	Area (95% CI)	Area (95% CI)	Area (95% CI)
Distressed Communities Index	0.531 (0.530-0.532)	0.479 (0.477-0.480)	0.511 (0.498-0.525)	0.499 (0.496-0.502)	0.531 (0.530-0.532)
Race/Ethnicity	0.469 (0.468-0.470)	0.403 (0.402-0.403)	0.471 (0.462-0.480)	0.473 (0.471-0.475)	0.481 (0.481-0.482)
Insurance	0.297 (0.296-0.297)	0.291 (0.290-0.292)	0.436 (0.425-0.446)	0.391 (0.389-0.393)	0.386 (0.385-0.386)
Household Median Income	0.464 (0.463-0.465)	0.510 (0.509-0.511)	0.489 (0.478-0.499)	0.502 (0.500-0.504)	0.469 (0.468-0.470)

Risk of heart failure

The predictive power of these variables, as evaluated by the ROC curve shown in Table 4, varied significantly. The DCI showed moderate predictiveness (AUC = 0.531, 95% CI 0.530-0.532), indicating a slight ability above random chance to discriminate heart failure occurrence. However, the predictiveness for race (AUC = 0.469, 95% CI 0.468-0.470), insurance type (AUC = 0.297, 95% CI 0.296-0.297), and household income quartiles (AUC = 0.464, 95% CI 0.463-0.465) was poor, demonstrating limited utility in predicting heart failure presence using these socioeconomic metrics alone. 

Atrial fibrillation 

The predictive power of patient race/ethnicity and the selected socioeconomic metrics was limited, as indicated by the area under the ROC curve: DCI (AUC = 0.479, 95% CI 0.477-0.480), race (AUC = 0.403, 95% CI 0.402-0.403), insurance type (AUC = 0.291, 95% CI 0.290-0.292), and household median income (AUC = 0.510, 95% CI 0.509-0.511) all showed poor discriminative ability to predict AF following HF-related admissions. This emphasizes the complexity of factors influencing AF in heart failure patients beyond simple socioeconomic and demographic metrics. 

Mortality following heart failure

For the risk of in-hospital mortality following heart failure-related admissions, race/ethnicity and socioeconomic factors showed generally low discriminative ability. The AUC for the DCI score was 0.499, with a 95% CI 0.496-0.502, indicating no better prediction than random chance. Similarly, low AUC values were observed for race (AUC = 0.473, 95% CI 0.471-0.475), insurance type (AUC = 0.391, 95% CI 0.389-0.393), and household median income quartiles (AUC = 0.502, 95% CI 0.500-0.504), suggesting the limited utility of these metrics alone in predicting in-hospital mortality among heart failure patients. 

Cardiac arrest 

Among individuals with heart failure who developed cardiac arrest, the overall predictiveness of race/ethnicity and the selected socioeconomic factors was generally low. The ROC curve for DCI was (AUC = 0.511, 95% CI 0.498-0.525), race (AUC = 0.471, 95% CI 0.462-0.480), insurance type (AUC = 0.436, 95% CI 0.425-0.446), and household median income (AUC = 0.489, 95% CI 0.478-0.499) suggested limited discrimination ability, indicating that these factors alone are not strong predictors of cardiac arrest among heart failure patients. 

Prolonged hospital stays 

For individuals who experienced prolonged hospital stays following a heart failure-related admission, the predictive power of race/ethnicity and the selected socioeconomic metrics was also generally low, as indicated by the ROC curve for DCI (AUC = 0.531, 95% CI 0.530-0.532), suggesting modest ability to predict prolonged stays. Similarly, race (AUC = 0.481, 95% CI 0.481-0.482), insurance type (AUC = 0.386, 95% CI 0.385-0.386), and household median income quartiles (AUC = 0.469, 95% CI 0.468-0.470) all demonstrated limited predictive capabilities, indicating that these factors alone do not strongly predict prolonged hospital stays in heart failure patients. 

## Discussion

Heart failure-related hospital admissions 

Our analysis of heart failure-related hospital admissions in Maryland (2016-2020) reveals significant disparities across race/ethnicity and socioeconomic metrics. Black individuals had a 33% higher risk of HF admissions, aligning with existing literature indicating increased HF incidence in this population. Conversely, Hispanic, Asian/Pacific Islander, Native American, and Other races showed lower or non-significant risk compared to White individuals. Medicare and Medicaid beneficiaries also exhibited higher odds of HF admissions, while private insurance was protective. Income levels were inversely related to HF admission risk, with individuals in the highest income quartile having 19% lower odds compared to those in the lowest income quartile. Additionally, individuals from distressed communities had a 12% higher risk of HF admissions compared to those from prosperous areas. These findings highlight socioeconomic and racial disparities in HF-related hospital admissions.

Mortality following heart failure

The association between race/ethnicity, socioeconomic status, and in-hospital mortality in Maryland reveals complex dynamics, particularly regarding the DCI and racial differences. Our findings indicate that patients from distressed zones and Asian/Pacific Islander individuals face higher mortality odds, a phenomenon not thoroughly captured in earlier literature. In contrast, Black patients showed unexpectedly lower mortality odds. Previous studies have shown mixed results; some indicate higher mortality for Black patients, while others, like ours, suggest a protective effect, potentially due to community or hospital-level factors not captured by broad socioeconomic indicators [16-19]. The strikingly high mortality odds in the "Others" insurance category could reflect inconsistencies in health care access or quality among less common insurance types, an area needing further exploration given its deviation from other findings where government insurance typically correlates with higher mortality.

Atrial fibrillation (AF)

Our study highlights significant racial disparities in the incidence of AF among heart failure patients, with lower odds for Black, Hispanic, and Asian/Pacific Islander patients compared to White patients. This contradicts the prevalent assumption in the literature that AF is less common in these racial groups due to underdiagnosis or less access to healthcare facilitating early detection [20]. The higher incidence of AF in higher-income quartiles suggests that socioeconomic factors may influence the recognition or management of AF symptoms [21]. The poor predictive power of socioeconomic metrics in our ROC analysis (AUC values below 0.5) underscores the limitations of using these alone for predicting AF, suggesting that other unmeasured variables like genetic predispositions or lifestyle factors might play significant roles.

Cardiac arrest

The findings regarding cardiac arrest in heart failure patients show minimal association with socioeconomic or demographic variables, indicating that the immediate triggers and risk factors for cardiac arrest may be more strongly influenced by acute medical conditions rather than long-term socioeconomic status. The significantly higher odds for Asian/Pacific Islander individuals might reflect genetic or culturally specific health dynamics that are not well documented in the current literature. Our results align with studies suggesting that socioeconomic metrics are poor predictors of acute events like cardiac arrest, which are more likely influenced by immediate clinical factors [22].

Prolonged hospital stay

The analysis related to prolonged hospital stays reveals a clear impact of the DCI, with patients from distressed zones more likely to experience more extended hospital stays. This might reflect greater disease severity or fewer resources for post-discharge care in these communities, aligning with findings from other studies highlighting how lower socioeconomic status contributes to worse overall health outcomes [23]. The limited impact of income quartiles suggests that while general economic status is a factor, the specific community context, as captured by the DCI, may play a more critical role in determining the length of hospital stays.

Limitations

Our study, focused on heart failure outcomes in Maryland, faces limitations, including its regional focus, which may not generalize globally, potential data incompleteness, and the simplistic measurement of socioeconomic status primarily through income and the Distressed Communities Index. Unmeasured confounders such as lifestyle choices and healthcare quality, the inability to infer causality from observational data, limited statistical power in subgroup analyses, and potential misinterpretation of odds ratios also challenge the robustness of our findings. Addressing these gaps in future research is vital for developing more precise interventions and understanding the complex influences on heart health.

## Conclusions

Overall, our findings highlight the nuanced roles of various socioeconomic metrics in predicting different health outcomes in heart failure patients. While socioeconomic status does influence health outcomes, the predictiveness of these metrics varies significantly across different conditions and outcomes. This study emphasizes the need for multifaceted healthcare policy and patient management approaches, considering both socioeconomic indicators and clinical factors to improve heart failure outcomes in diverse populations. The limited predictiveness of traditional socioeconomic metrics highlights the necessity for integrating more specific social and clinical data into patient care strategies to tailor interventions more effectively and improve health outcomes across different demographic and socioeconomic groups.
